# The incidence of left atrial appendage thrombi on transesophageal echocardiography after pretreatment with apixaban for cardioversion in the real-world practice

**DOI:** 10.1371/journal.pone.0208734

**Published:** 2018-12-07

**Authors:** Jongmin Hwang, Hyoung-Seob Park, Seung-Woon Jun, Sang-Woong Choi, Cheol Hyun Lee, In-Cheol Kim, Yun-Kyeong Cho, Hyuck-Jun Yoon, Hyungseop Kim, Chang-Wook Nam, Seung-Ho Hur, Sang Hoon Lee, Seongwook Han

**Affiliations:** 1 Department of internal medicine, Division of Cardiology, Keimyung University Dongsan Medical Center, Keimyung University School of Medicine, Daegu, Republic of Korea; 2 Department of internal medicine, Division of Cardiology, Department of Internal Medicine, SM Christianity Hospital, Pohang, Republic of Korea; Ziekenhuisgroep Twente, NETHERLANDS

## Abstract

The risk of thromboembolisms during the post-cardioversion period is high. For patients with persistent atrial fibrillation (AF), anticoagulation with warfarin (INR 2.0~3.0) is recommended for at least three weeks prior and four weeks after cardioversion. We aimed to evaluate the efficacy of apixaban in preventing thromboembolic events during post-cardioversion. We enrolled 127 consecutive persistent AF patients (83 persistent, 44 longstanding persistent AF), scheduled to undergo cardioversion and were pretreated with apixaban. All patients underwent transesophageal echocardiography (TEE) to rule out thrombi in the left atrium (LA) or LA appendage (LAA) after anticoagulation with apixaban. The median duration of anticoagulation before the TEE was 37 (interquartile range [IQR] 34, 50) days. There were 7 patients (5.5%) with visible thrombi in the LAA. A spontaneous echo contrast was noted in 24 (18.9%) patients. Cardioversion was attempted in 117 patients, and they were prescribed amiodarone before the elective DC cardioversion. Sinus rhythm was achieved in 37 patients (31.6%) by amiodarone itself. DC cardioversion was attempted in 80 patients and was successful in 73 (91.3%). None of the cardioverted patients had any thromboembolic events within one month. Transient ischemic attacks were observed in one patient during a median follow up period of 202 days (IQR 143, 294). In conclusion, apixaban could be used as an anticoagulant for patients scheduled for cardioversion. However, the incidence of thrombi was not negligible. TEE or other imaging modalities should be considered before cardioversion or other invasive procedures.

## Introduction

Rhythm control therapy is an integral part of atrial fibrillation (AF) management. It is performed to improve symptoms in AF patients who remain symptomatic on adequate rate control therapy [1]. For rhythm control therapy in persistent AF patients, pharmacologic cardioversion or elective synchronized direct current electrical cardioversion after adequate anticoagulation is performed. However, restoring sinus rhythm contains the risk of thromboembolisms during the post-procedural period. Without preceding anticoagulation, cardioversion carries a 5–7% risk of thromboembolic complications [2]. This thromboembolic risk is also high after successful cardioversion, especially in patients with conventional risk factors for thrombo-embolism [3]. Therefore, the guidelines recommend adequate anticoagulation for at least three weeks before and four weeks after cardioversion in patients with AF of longer than 48 hours or unknown duration. The vitamin K antagonist, warfarin, was traditionally used for thromboembolic prophylaxis in this situation but non-vitamin K antagonist oral anticoagulants (NOACs) also showed comparable low post-cardioversion stroke risk [4–6]. In this study, we investigated persistent AF patients who underwent anticoagulation for elective cardioversion using apixaban, one of the NOACs. Through the TEE findings before the cardioversion in these patients, we sought to evaluate the anticoagulant effect of apixaban in the real clinical setting.

## Methods

### Study population and baseline assessment

We enrolled consecutive patients who presented with persistent or long-standing persistent AF and agreed to undergo cardioversion at our hospital from September 2014 to March 2018. Persistent AF was defined as continuous AF lasting more than seven days. Long-standing persistent AF was defined as continuous AF lasting more than one year. Baseline demographic and clinical data were collected, and the CHA_2_DS_2_-VASc scores were calculated for each patient. Conventional transthoracic echocardiography (TTE) was performed to rule out any structural heart disease including valvular heart disease. All patients signed their informed consent for the procedure and the study was approved by the research ethics committees of the institution (institutional review board of Dongsan Medical Center (Number: 2015-08-014)).

### Anticoagulation

Patients without moderate to severe mitral valvular heart disease and that agreed to the rhythm control strategy were anticoagulated with apixaban for at least three weeks before the attempt of cardioversion. The apixaban was administered with a 5-mg twice daily dose. The 2.5-mg twice-daily dose was used in two patients who had two or more of the following criteria: an age of more than 80 years, body weight of less than 60 kg, or serum creatinine level of 1.5 mg per deciliter or more.

### Transesophageal echocardiography

The transesophageal echocardiography (TEE) was performed after at least three weeks of apixaban medication. The TEE was performed using a 5–7 MHz broadband, multiplane transducer (Vivid Q, GE healthcare, Solingen, Germany). The presence of a spontaneous echo contrast (SEC) and left atrial (LA) and LA appendage (LAA) thrombi was confirmed by the two attending physicians without knowing the clinical information of the patient. A SEC was defined as dynamic smoke-like echoes within the atrial cavity, with a characteristic swirling motion that could not be eliminated by changes in the gain settings. The severity of the SEC was graded according to the following criteria: “Mild” was defined as minimal echogenicity located in the LAA or sparsely distributed in the main cavity of the LA, which was possible to detect only transiently during the cardiac cycle, but imperceptible at operating gain settings for the echocardiographic analysis. “Moderate” was defined as a dense, swirling pattern in the LAA, generally associated with a somewhat lesser intensity in the main cavity, which may fluctuate in intensity, but detectable constantly throughout the cardiac cycle. “Severe” was defined as an intense echo density and a very slow swirling pattern in the LAA, usually with a similar density in the main cavity [7–10]. A thrombus was considered to be present if a mass detected in the LAA or in the LA cavity appeared to be distinct from the underlying endocardium, was not caused by pectinate muscles, and was detected in more than one imaging plane [9, 10].

### Cardioversion and follow-up

The patients who had thrombi in the LA or LAA were not intended to undergo cardioversion. In the patients with an SEC, whether to perform a cardioversion was judged by the attending physician. The patients who did not have any thrombi or SECs on the TEE were prescribed amiodarone for at least a month with a loading dose expecting a chemical cardioversion and prevention of immediate recurrences of AF after the electrical cardioversion. The electrical cardioversion was performed if the patient did not restore sinus rhythm with amiodarone alone. All cardioverted patients were continued on amiodarone and apixaban for at least one month afterward. Follow-up clinical data to assess for any strokes, transient ischemic attacks, or systemic embolic events occurring within 30 days after the cardioversion were collected.

### Statistical analysis

Continuous variables are expressed as the mean value ± standard deviation or median values (interquartile range [IQR]: 25th percentile–75th percentile). Categorical variables are expressed as numbers and percentages. All statistical analyses were performed using the MedCalc software package, version 18.5 (MedCalc Software, Mariakerke, Belgium). A P value <0.05 was considered statistically significant.

## Results

### Patient clinical characteristics

A total of 127 patients were enrolled. The mean age was 57.9 ± 8.4 years, and 104 patients (81.8%) were male. Of those, 83 (65.4%) were persistent AF patients, and the median duration of AF was 89 (IQR 50, 232) days. The remaining 43 patients were long-standing persistent AF patients. The mean CHA_2_DS_2_-VASc score of the enrolled patients was 1.0 ± 1.2. The detailed baseline characteristics of the patients are summarized in Table 1.

**Table 1:** Baseline characteristics.

Characteristic	Total N = 127
Male	104 (81.8)
Age (y)	57.9 ± 8.4
Type of AF	
Persistent	83 (65.4)
Long-standing persistent	44 (34.6)
CHA₂DS₂-VASc score	1.0 ± 1.2
Score 0–1	92 (72.4)
Score ≥ 2	35 (27.6)
Patients with LV EF <40%	8 (6.4)
History of hypertension	37 (29.6)
History of diabetes	15 (12)
History of stroke/TIA/thromboembolism	6 (4.8)
History of vascular disease	3 (2.4)
Serum creatinine	0.9 ± 0.2
†Duration of anticoagulation before TEE (days)	37 (34, 50)
Echocardiographic findings	
LV EF	57.2 ± 10.5
LA volume (ml)	106.0 ± 33.1
LA volume index (ml/m^2^)	57.6 ± 17.8
SEC on TEE	24 (18.9)
Mild	15 (11.8)
Moderate to severe	9 (7.1)
Thrombus on TEE	7 (5.5)

Values are presented as n (%) or mean ± SD. †Duration of anticoagulation is presented as median (interquartile range). AF: atrial fibrillation, LV: left ventricle, EF; ejection fraction, TIA; transient ischemic attack, LA: left atrium, SEC; spontaneous echo contrast, TEE; transesophageal echocardiography.

### TEE findings

All patients underwent TEE before the cardioversion. The median duration of the anticoagulation with apixaban before the TEE was 37 (IQR 34, 50) days. Among 127 patients, 24 (18.9%) had an SEC during the TEE. Nine patients had a moderate SEC and 15 a mild SEC. No severe SECs were observed. Thrombi were observed in 7 (5.5%) patients. Five patients had multiple or single small thrombi, and two patients had mural thrombi in the LAA. The characteristics of the patients with LAA thrombi are summarized in Table 2.

**Table 2:** Characteristics of patients with LAA thrombus.

Patient No.	Age	Sex	Type	CHA_2_DS_2_-VASc	LV EF	LA VI	SEC	Duration of Apixaban Tx (days)	Thrombus feature
1	64	F	Persistent	4	31	63.2	No	58	Multiple tiny thrombi
2	52	M	Persistent	0	60	52.0	Yes	27	0.5 cm, single thrombus
3	66	F	Longstanding persistent	3	65	81.8	Yes	45	Multiple tiny thrombi
4	63	F	Longstanding persistent	1	70	59.7	Yes	52	Multiple tiny thrombi
5	74	M	Persistent	4	16	58.2	No	30	LAA Mural thrombi
6	67	F	Persistent	4	55	78.4	Yes	75	LAA Mural thrombi
7	67	M	Persistent	1	40	52.4	Yes	25	Multiple tiny thrombi

LV EF: left ventricular ejection fraction, LA VI: left atrial volume index, SEC: spontaneous echo contrast, Tx: treatment

We performed uni- and multivariate regression analyses to assess which clinical variables were associated with thrombus on the TEE. In the univariate analysis, an age over 65 years old, female sex, CHA_2_DS_2_-VASc ≥ 3, left ventricular ejection fraction (LV EF) ≤ 40% were significantly associated with thrombi. However, in the multivariate analysis, only an LV EF ≤ 40% was independently associated with thrombi. A female sex and CHA_2_DS_2_-VASc ≥ 3 also exhibited a marginal significance. The median duration of the anticoagulation did not differ between patients with and without thrombi. This is summarized in Table 3.

**Table 3:** Clinical variables associated with left atrial thrombus.

Variable	Univariate regression		Multivariate regression	
	OR (95% CI)	P-value	OR (95% CI)	P-value
Age > 65 years	6.67 (1.38–23.11)	0.018	2.22 (0.25–19.55)	0.474
Female sex	7.02 (1.45–33.91)	0.015	7.44 (0.83–66.61)	0.073
Longstanding PeAF	0.74 (0.14–3.99)	0.729	0.85 (0.10–7.05)	0.880
CHA_2_DS_2_-VASc ≥ 3	13.21 (2.61–66.78)	0.002	9.94 (0.89–110.45)	0.062
EF ≤ 40%	14.25 (2.58–78.58)	0.002	17.95 (1.59–202.90)	0.020
LA VI ≥ 60 mL/m^2^	1.13 (0.24–5.25)	0.881	0.33 (0.03–3.69)	0.372
Duration of anticoagulation	Thrombus (+) vs (-): median 45 vs 37 days (M-W test, p = 0.891)			

OR: odds ratio, CI: confidence interval, PeAF: Persistent atrial fibrillation, EF: left ventricular ejection fraction, LA VI: left atrial volume index, M-W test: Mann-Whitney test.

### Cardioversion and Follow-up

**Fig 1:**
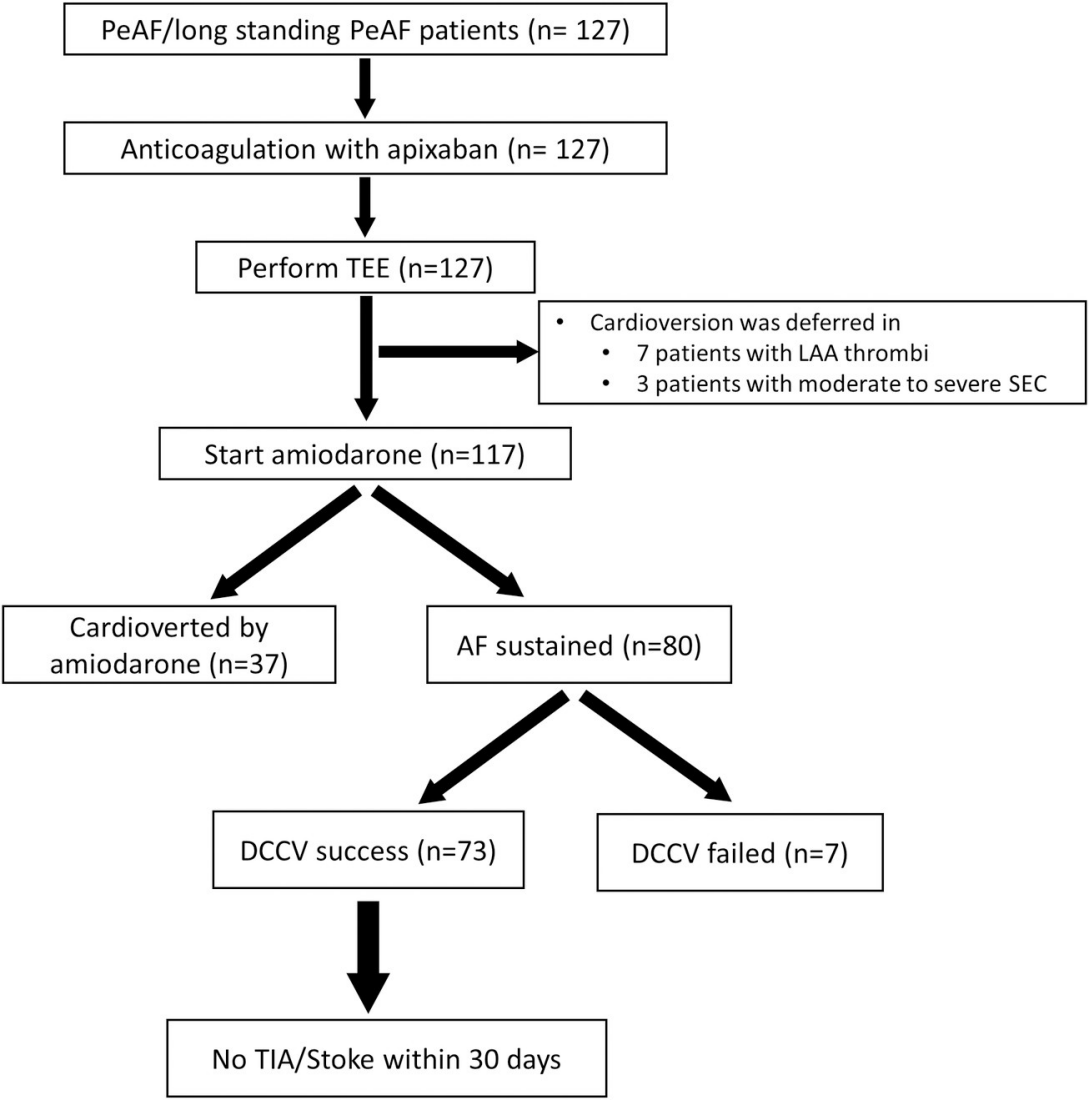
Flow chart of the cardioversion. This figure shows our study design and the results of the cardioversion. PeAF: persistent atrial fibrillation, TEE: transesophageal echocardiography, LAA: left atrial appendage, SEC: spontaneous echo contrast, DCCV: direct current cardioversion, SR: sinus rhythm.

All seven patients with thrombi in LAA were deferred cardioversion and continued anticoagulation treatment. For patients with an SEC, cardioversion was performed in 19 patients and was deferred in 3 patients. Of the 117 patients in whom cardioversion was attempted, 37 (37/117, 31.6%) were cardioverted by amiodarone. Electrical cardioversion was performed in 80 patients who had AF despite amiodarone administration, and 73 (73/80, 91.3%) were successfully restored to sinus rhythm. In those patients, 30 (30/73, 41.1%) remained in sinus rhythm after a median follow up period of 202 days (143, 294). The treatment flow and results of the cardioversion are described in Fig 1.

### Thromboembolic or serious adverse events

None of the cardioverted patients had any strokes or systemic embolism events within one month or during the follow up period after the cardioversion. One patient who had recurrent AF after the cardioversion had a transient ischemic attack that occurred 45 days after the cardioversion. No other serious adverse events were observed.

## Discussion

In this study, 5.5% of the persistent AF patients had LAA thrombi, and 18.9% had an SEC in the LA during the TEE after a median of 34 days on apixaban medication for cardioversion. Although it was not a large number of patients, it was notable that thrombi were observed in 5.5% of the patients, despite the five weeks anticoagulant therapy, which was two weeks longer than the recommended anticoagulant therapy period suggested by the current guidelines.

Performing cardioversion has the potential of thromboembolisms [3], which are reduced substantially by the administration of anticoagulation [11]. Patients who have been in AF for longer than 48 hours should start oral anticoagulation and be maintained on that for at least 3 weeks before a cardioversion and continue that for 4 weeks after the cardioversion. Further, it should then be continued indefinitely in patients at risk of a stroke [1]. Analyses from the RE-LY (dabigatran) [12], ROCKET-AF (rivaroxaban) [13], and ARISTOTLE (apixaban) [14] trials suggest that electrical cardioversion in patients treated with NOACs has a similar (and very low) thromboembolic risk as that under warfarin. Later prospective trials with rivaroxaban (X-VeRT) [4], edoxaban (ENSURE-AF) [5], and apixaban (EMANATE) [6] have confirmed the low post-cardioversion stroke risk in patients treated with an NOAC. Based on the results of these studies, it is now considered safe to perform cardioversion without a TEE in people taking NOACs for over three weeks.

However, there is no coagulation assay for NOACs, and an adherence assessment of patients for NOACs has inherent limitations. Therefore, there is always concern about whether it is safe to perform cardioversion without a TEE. Although some studies have shown that the incidence of LA thrombi in patients treated with NOACs is zero or very low [15, 16], some studies have not. Frenkel et al. reported that LA thrombus rates detected by TEE among patients on dabigatran, rivaroxaban, and apixaban, were 5.4%, 4.8%, and 0%, respectively [17]. Wu et al. reported that in patients on continuous NOAC therapy, 2.8% of LA thrombi and 2.5% of dense SECs were detected by TEE prior to AF ablation [18]. The results of these studies are consistent with our findings.

Previous studies showed that the presence of LA/LAA thrombi increases significantly with higher CHADS_2_ scores despite appropriate anticoagulation with warfarin [19, 20]. In the era of NOACs, similar results have also been reported [18, 21]. In our analysis, the LVEF was independently associated with LA thrombi. Further, a female sex and CHA_2_DS_2_-VASc score over 3 also showed a marginal statistical significance. Therefore, if the need for TEE is to be assessed, conventional risk factors such as congestive heart failure or CHA_2_DS_2_-VASc scores may be helpful.

### Limitations

First, our study was a single center prospective observational study with a small number of patients. Second, only apixaban was assessed among the 4 available NOACs. At the time of designing the study, the X-VERT study using rivaroxaban was published [4]. The national insurance coverage for edoxaban was initiated after the study had begun. Hence, we sought to evaluate the efficacy of apixaban in the real-world practice. Third, the enrolled patients mainly consisted of persistent AF patients and received amiodarone as a pretreatment for elective electrical cardioversion. For the maintenance of sinus rhythm after cardioversion, amiodarone is known to be better than a placebo [22, 23], beta-blockers [23], sotalol [22, 24], propafenone [24], or other class I agents [25]. Based on those references, we decided to use amiodarone as a pretreatment for elective cardioversion, which is more effective than other antiarrhythmic agents for sinus rhythm maintenance and is known to help diminish the immediate recurrence of AF after DCCV.

## Conclusion

In conclusion, our study showed that anticoagulation using apixaban was feasible. However, it also revealed that thrombi and an SEC in the LA/LAA were not negligible in adequately anticoagulated patients. Therefore, it is reasonable to consider TEE or other imaging modalities to rule out LA/LAA thrombi in patients undergoing cardioversion or more invasive procedures, such as catheter ablation of AF. The development of a risk stratification system for the need for imaging to rule out LA/LAA thrombi is warranted by a large-scale study.

## Supporting information

S1 Table Individual-level data underlying results presented in this article. (XLSX)
